# Water soluble curcumin with alkyl sulfonate moiety: Synthesis, and anticancer efficacy

**DOI:** 10.1016/j.heliyon.2024.e33808

**Published:** 2024-06-28

**Authors:** Alaa Janem, Ghader Omar, Othman Hamed, Shehdeh Jodeh, Abdalhadi Deghles, Avni Berisha, Waseem Mansour, Saber Abu Jabal, Oswa Fares, Ataa Jaser, Ameed Amireh, Ghaleb Adwan

**Affiliations:** a Chemistry Department, Faculty of Science, An-Najah National University, P.O. Box 7, Nablus, Palestine; b Biology Department, Faculty of Sciences An-Najah National University, P.O. Box 7, Nablus, Palestine; c Department of Chemistry, Istiqlala University, Jericho, Palestine; d Department of Chemistry, Faculty of Natural and Mathematics Science, University of Prishtina, Prishtina, 10000, Republic of Kosovo; e Materials Science-Nanochemistry Research Group, Nano Alb-Unit of Albanian Nanoscience and Nanotechnology, Tirana, 1000 Albania

**Keywords:** Curcumin, Anticancer, Molecular docking, Sultone, Hela cells

## Abstract

Curcumin is classified as a chemotherapeutic medication because of its potential against numerous cancer cell lines and ability to inhibit cancer cell proliferation. Despite these findings, curcumin has yet to be commercialized as a drug due to its low water solubility, low absorption, and restricted bioavailability. As a result, there is a demand for water-soluble curcumin with improved solubility, bioavailability, and thus bioactivity. In this study we report the synthesis and the anticancer activities of water-soluble curcumins derivatives with alkyl sulfonate moiety. The target water-soluble curcumin with alkyl sulfonate moieties was created utilizing a straightforward technique that involved reacting curcumin with various sultones. The cytotoxic (24 h) and cytostatic (72 h) anticancer effect on breast carcinoma (MCF-7), liver carcinoma (HepG2), skin melanoma (B16–F110), colon human cancer and HeLa cervical carcinoma cell lines viability % via MTT assay were determined for the prepared derivatives. Results showed that curcumin-derived compounds have a pronounced cytostatic anticancer effect rather than cytotoxic one in relation to the compound type, cancer cell line type, and examined concentration compared to curcumin. The curcumin sulfonates outperformed curcumin activity against the tested cancer cells and showed to be powerful anticancer candidate drugs as supported by the theoretical calculations. This is evident by their high capacity to form H-bonding during docking with the amino acid side chains and the Vina docking score.

## 1 Introduction

Cancer will have surpassed all other causes of mortality globally by 2030, according to the World Health Organization, which predicts a 69 % increase, or 21 million more cases. Both the general increase in the world's population and the aging of the population are major contributors to the alarming rise in cancer rates [1,2]. As on the most recent cancer mortality reports, these causes were responsible for 14.7 % of all deaths in 2017 [3,4].

Despite breakthroughs, drug resistance and side effects still hinder cancer detection, treatment, and prevention. While former kills, latter lowers cancer therapy efficacy [5,6]. Researchers have focused on biobased therapeutics with the highest curative results and lowest side effects [7]. Because of their diversity and uniqueness, biobased products are useful in the quest for novel drugs, especially in the fight against cancer and infectious diseases, which can be devastating [8,9].

One of the most interesting biobased products is curcumin (1,7-bis(4-hydroxy-3-methoxyphenyl)-1,6-heptadiene-3,5-dione), which has numerous commercial and medicinal uses, including in nutraceuticals, cosmetics, and pharmaceuticals [10]. To top it all off, it's safe to use [11] and has a wide range of bioactivities, such as fighting malaria [12], cancer [13,14], microbes [14,15], antioxidants [14], diabetes [16], and bacteria [17]. Among the aforementioned bioactivities, curcumin was found to exhibit apoptosis potential chemotherapeutic drug, as can restrain the propagation of different cancer cells growth [[18], [19], [20], [21]] and improve patients' quality of life by minimizing radiation and chemotherapy side effects. Reason being, curcumin controls the activity of angiogenic growth factors and transcription factors, and it suppresses the expression of genes implicated in tumour angiogenesis and invasion [22].

**Fig. 1:**
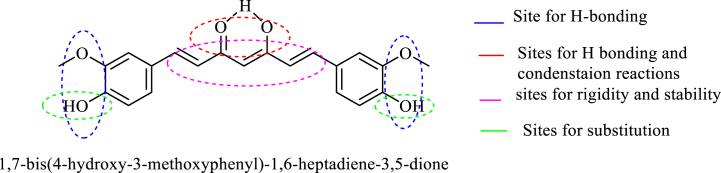
Active sites in curcumin structures.

Despite all these unique properties curcumin has not been industrialized as a commercial drug due to its poor solubility, low absorption, and limited bioavailability [[23], [24], [25]]. The hydrophobicity of curcumin causes it to penetrate the cell membrane and bind to the fatty acyl chains of membrane lipids through hydrogen binding and hydrophobic interactions, resulting in low cytoplasmic curcumin availability [26,27]. The aryl part of the ring, the olefin chain, the diketo functionality, and the methylene site are the four primary sites that make up the unique structure of curcumin (Fig. 1) [21,23].

To study the associated change in stability and activity of the curcumin compound, numerous analogs and derivatives have been developed that target each of the mentioned above sites [23,24]. According to a published work, the diketo moiety is responsible for curcumin's instability and rapid in vivo metabolism, and it must be addressed by including scaffolds that can increase curcumin's effectiveness. In a study of the structure-activity relationship of curcumin's diketone chain, Reddy et al. hypothesized that adding hydrazine results in derivatives with more rigid structure should enhance the anticancer activity [25]. Additionally, Jankun et al. have postulated another study that converting the middle part to a ring could lead to curcumin with enhanced bioactivity [30].

Hence, new phytochemical-inspired polymer prodrugs have been produced by chemical modification and loading into micro and nanostructures with several biological benefits via combining adjuvants, encapsulating in carriers, and manufacturing in nanoforms with other bioactive compounds, synthetic derivatives, and structural analogs [28]. For example, utilizing biotechnology and nanotechnology to address curcumin's disadvantages might increase its medicinal uses and clinical efficacy [29].

Moreover, water insolubility of curcumin, instability limitation and poor bioavailability were enhanced by approaches [31] like development of micellar and lipid-drug hybrid nanoparticles [32]. However, the toxicity issues as well as limited passage of the micelles and slow drug release of this hybrid cannot be disregarded [24,25,30,[33], [34], [35]]. Bridging curcumin at the β-diketone site with hydrazine moiety may change its molecular structure and enhance bioactivity by making it more likely to interact with lipid membranes or other hydrophobic environments. Unfortunately, β-diketone functionality when bridged with various hydrazines made curcumin more hydrophobic [31,36]. Nevertheless, hydrophobicity effects vary for each β-diketone and hydrazine derivative, depending on its kind, incorporation into curcumin, and impact. These changes in curcumin structure must be carefully designed and characterized to understand their hydrophobicity profiles and biological effects [36].

Continuous improvement of the possibility of using curcumin as an anticancer adjuvant with kidney-protective effects would increase the efficacy of cisplatin and safety in clinical setting [37]. The effects of nanocurcumin on its pharmacokinetic parameters in blood and other organs were also studied as another approach for enhancing curcumin effectiveness and bioavailability, finding no enhancement in both plasma and organs, except for ovaries [38].

To further enhance curcumin's water solubility, a water-soluble nano-formulation was synthesized using two acid-responsive polymeric micelles that included both covalent and non-covalent curcumin. This was achieved by combining curcumin with the biodegradable copolymer methoxy polyethylene glycol-polylactic acid (PEG-PLA), which allowed for the formation of two conjugates with distinct topologies (single-tail and double-tail, respectively). Though the two micelles had identical hydrodynamic dimensions, the single-tailed one had a greater loading capacity, resulting in quicker release and lower IC50 against HeLa cells. This nanoplatform successfully distributes large hydrophobic molecules (curcumin) intracellularly in response to stimuli, boosting its therapeutic benefits [39]. Likewise, a different nanoparticles formulation of curcumin successfully inhibited the growth of colon-26 carcinoma cells through binding and cellular absorption 19 times more effectively than free curcumin [40]. In addition, curcumin was added to myristic acid-chitosan nanogel-based nanoparticles to improve its anticancer effects, by nano's drug-targeting [41]. Also, compared to other nanoparticles, curcumin showed enormously improved absorption in the prostate cancer cell line PC3 as well as the human embryonic kidney cell line HEK [42]. The use of silk fibroin nanoparticles caused the curcumin to be released more slowly into colon cancer cells (HCT116), which reduced its cytotoxic effects on healthy cells [43].

Thereby, the application of nanoscale drug delivery system technologies as nanocarriers was implemented, resulting in improved solubility, stability, simplification of preparation, dosage reduction, and the mitigation of adverse effects [44]. Thus, curcumin bioavailability issues were resolved through the application of nanotechnology via wet milling causing significantly three times higher antiproliferative potency therapeutic effect of the nanoparticles than that of free curcumin by in-vitro testing on lung, liver, and skin cancer [45]. Recent developments in nanomedicine have led to the creation of solid lipid nanoparticles with large therapeutic payloads, extended shelf lives, biocompatibility, and ease of manufacturing. One such example is the creation of lipidic nano-constructs encapsulated with curcumin [46].

Groundbreaking research by altering the drug transport and therapeutic effects against breast cancer, innovative ultralong-circulating paclitaxel nanoparticles coated with curcumin derivatives were developed, paving the way for an optimization of the widely used anticancer medication paclitaxel. So, curcumin derivatives provide a new level of excitement to nanomedicine through its incorporation [47]. Furthermore, Pegylated curcumin derivatives were produced by directly esterifying PEG 600 with curcumin in the presence of N,N′-dicyclohexyl carbodiimide. They were versatile due to their excellent water solubility along with good antitumor activity against Grafficell cancer cell lines at a relatively low active concentration in relation to the amount of curcumin in the conjugation and the incubation duration [48].

Into the bargain, curcumin has been functionalized with a single methacrylate group to create a monomer termed MMC, which is another technique to enhance its limited solubility and quick breakdown in water [49]. Additionally, polymeric micelle approach cytotoxicity concentration-based water-soluble curcumin nano-formulation using chito-oligosaccharides and pluronic F-68 showed promise as a potential drug delivery mechanism, allowing scientists to circumvent the low curcumin water solubility restriction enhancing suppression of NO release activity and considerably decreased cytotoxicity of leukemia cells as compared to natural curcumin [50]. The persistent endeavors of several researchers to enhance the water solubility of curcumin with nanoparticles-curcumin conjugation (such polysaccharide or silica) as cyclodextrin complexes and nanosuspensions were two other possibilities [51].

Furthermore, curcumin cod protein encapsulation was adopted to improve solubility and stability through a pH-driven mechanism which has the makings of a fantastic edible curcumin encapsulant [52]. Similarly, food protein fibrils are progressively recognized as promising matrixes for the preservation and delivery of bioactive compounds which were utilized as carriers to complex with curcumin. This curcumin modification presented excellent antioxidant and outstanding sustained-release properties, offering further opportunities for the release of curcumin in the intestine and improving its bio-accessibility and bioavailability [53].

In our perspective, curcumin and its scaffolds have the potential to improve bioavailability, toxicity, and selectivity for certain malignancies. The present pivotal study might be focused on synthesizing curcumin derivatives that overcome the drawbacks and improve the bioactivity on this platform. Prior to doing pre-clinical validation in animal models and clinical evaluation in humans, cancer cell-based assays are crucial for studying chemotherapeutic efficiency and mechanisms [54]. As part of the process of researching a potential novel medicine, cell culture tests are used to assess the antiproliferative activity of cells.

Taking this into consideration, we provide here a study on the synthesis of ionic curcumin with sulfonate functionality, along with a comprehensive analysis of its effectiveness against a variety of cancer cell lines including skin melanoma (B16–F110), liver carcinoma (HEPG2), breast carcinoma (MCF-7), Colon human cancer and HeLa cervical carcinoma cell lines. The results were compared to the parent curcumin, promising results were obtained. The curcumin derivatives we present in this study are made from available low-cost materials using a very simple one step method. No special reaction conditions or special equipment are needed for making them.

## 2 Materials and methods

All prepared curcumin sulfonates were characterized by Nuclear Magnetic Resonance (NMR), FT-IR and MS/MS. Chemicals were obtained from Aldrich Chemical Company (Jerusalem) and used as is. NMR spectra were recorded on Varian Gemini instrument 2000, 300 MHz using dimethylsulfoxide-d_6_, ^13^C NMR peaks were reported in ppm relative to DMSO‑*d*_6_ (39.52 ppm). The FT-IR spectra were obtained using a Shimadzu spectrometer 820 PC FT-IR (Kyoto. Japan). The MS/MS analysis was performed using Thermo-Fisher Scientific LCQ Fleet ion trap mass spectrometer (CA, USA) that was run in a positive electrospray mode with a voltage of 5.0 kV, a capillary temperature of 295.0 °C, and a gas flow of 30.0 units. An isolation width of 2.0 Da with a 20.0 msec activation time was used for the M.S. method. All scans were acquired with an ionization-time of 250.0 ms. The purification of the prepared curcumin derivatives was performed by recrystallization.

### 2.1 Preparation of 3,3'-((((1E,3Z,6E)-3-hydroxy-5-oxohepta-1,3,6-triene-1,7-diyl)bis(2-methoxy-4,1-phenylene))bis(oxy))bis(propane-1-sulfonic acid) (1)

The reaction was carried out in a round-bottomed flask fitted with a refluxing condenser. A curcumin solution (**1**, 2.72 mmol, 1.0 g) in THF (30.0 mL) was prepared and treated with NaOH powder (0.212 g, 5.3 mmol). The produced suspension was mixed at room temperature for 20 min, then treated with 1,3-propanesultone (0.66 g, 5.4 mmol). The produced mixture was refluxed for 2.0 h. The produced solid was collected by suction filtration, rinsed with 5 % HOAc solution in methanol (2 x 30 mL) to remove residual sodium hydroxide and the third time with methanol (50 mL). The brown solid was recrystallized from a water-ethanol mixture (1:1 ratio by volume). Product mass 1.23 g (71.92 % yield). Melting point 237–240 °C. IR: *v*_max_ cm^−1^ 3451(O–H stretching), 1630(C=O, carbonyl), 1583(C=C, alkene),1512(C=C alkene), 1469(C=C benzene), 1135(S–O stretching), 1191(O=S=O stretching), 1048(C–O stretching). ^1^H NMR (300 MHz, DMSO‑*d*_6_) *δ*: 10.31, (bs, 1H, vinylic O–H), 7.55 (d, 1H, *J = 7.5,* aromatic), 7.25 (d, 1H, *J = 15.2* vinylic), 7.14 (m, 2H, aromatic), 7.04 (s, 1H, aromatic), 6.94 (s, 2H, aromatic), 6.84 (d, 1H, *J = 7.5,* aromatic), 6.73 (m, 2H, vinylic), 6.43 (s, 2H, sulfonic), 6.05 (s, 1H, vinylic), 4.12 (t, 4H, *J = 7.2* methylene), 3.87 (s, 6H, O–CH_3_), 3.05 (t, 4H, *J = 7.2.* methylene), 2.33 (m, 4H). C-13 (400 MHz, DMSO‑*d*_6_) *δ:* 183.7, 150.3, 142.1, 140.5. 133.6. 127.1. 123.5, 122.3, 119.2, 118.3, 111.4, 101.1, 67.6, 56.6, 48.2, 24.1. MS/MS [M + 1] for C_27_H_32_O_12_S_2_: Calculated 613.16, found: 613.72.

### 2.2 Preparation of 4,4'-((((1E,3Z,6E)-3-hydroxy-5-oxohepta-1,3,6-triene-1,7-diyl)bis(2-methoxy-4,1-phenylene))bis(oxy))bis(butane-1-sulfonic acid) (2)

The reaction was carried out in a round-bottomed flask fitted with a magnetic stirring bar and a refluxing condenser. A curcumin solution (**1**, 2.72 mmol, 1.0 g) in THF (30.0 mL) was prepared and treated with NaOH powder (0.212 g, 5.3 mmol). The produced suspension was stirred at room temperature for 20 min, then treated with 1,4-butanesultone (0.73 g, 5.4 momole). The reaction was then refluxed for 2.0 h. The produced solid was collected by suction filtration, rinsed with 5 % HOAc solution in methanol (2 x 30 mL) to remove residual sodium hydroxide and the third wash was with methanol (50 mL). The produced brown solid was recrystallized from a water-ethanol mixture (1:1 ratio by volume). The solid product was 1.17 g with brown color (yield 73.12 %). Melting point 275–276 °C. IR: *v*_max_ cm^−1^ 3451 (O–H stretching), 1630 (C=O, carbonyl), 1583(C=C, alkene),1512(C=C alkene), 1469 (C=C benzene), 1135 (S–O stretching), 1191 (O=S=O stretching), 1048 (C–O stretching). ^1^H NMR (300 MHz, DMSO‑*d*_6_) *δ*: 10.31, (bs, 1H, vinylic O–H), 7.57 (d, 1H, *J = 15.2,* vinylic), 7.51 (d, 1H, *J = 15.2,* vinylic), 7.14 (m, 2H, *J = 7.2,* aromatic), 7.06 (s, 1H, aromatic), 6.94 (m, 2H, *J = 7.2,* aromatic), 6.93 (s, 2H, sulfonic), 6.86 (d, 1H, aromatic), 6.75 (m, 2H, vinylic), 6.03 (s, 1H, vinylic), 4.1 (t, 4H, methylene), 3.86 (s, 6H, O–CH_3_), 3.02 (t, 4H, methylene), 1.92 (m, 8H).C-13 (400 MHz, DMSO‑*d*_6_) *δ:* 183.5, 150.1, 142.3, 140.4. 133.8. 126.9. 123.7, 122.2, 119.5, 118.6, 111.5, 101.3, 68.7, 56.3, 52.0, 27.8, 19.8. MS/MS [M + 1] for C_29_H_36_O_12_S_2_: Calculated 642.2, found: 642.46.

### 2.3 Anticancer testing

#### 2.3.1 Solutions preparation

The anticancer testing was performed on compounds 1 and 2 in sulfonate form to enhance the solubility in water. For MTT assay, 40 mg of compounds 1 and 2 were dissolved in up to 2.00 mL of freshly prepared RPMI media, and curcumin in freshly made 1 % fresh DMSO in RPMI media, to yield a stock solution of 2.00 mg/mL final concentration. Under aseptic conditions at the Sterilizer Biosafety cabinet, 0.25 μm membrane microfiltration was used to prepare sterilized diluted solutions with concentrations ranging from 31.25 to 1000.00 μg/mL through two-fold dilutions directly in 96 well microtiter plates.

#### 2.3.2 Cell lines and culture medium

B16–F10skin melanoma, HEPG2liver carcinoma, MCF-7breast carcinoma, Colon human cancer and HeLa cervical carcinoma cell lines used in this work were purchased from ATCC Culture Collection (USA). The cells were cultured in media in T25 cell culture flask from Roswell Park Memorial Institute (RPMI 1640). The media was freshly supplemented with 1 % v/v of penicillin-streptomycin (antibacterial effect), heat inactivated fetal bovine serum (FBS) (10 %), antifungal effect (1 % v/v) and l-glutamine (1 % v/v, as an energy source). Cancer cells were incubated completely isolated from light in a CO_2_ medium at 37.0 °C, 95.0 % humidity, 5 % CO_2_. The cells subjected to culturing were routinely checked under an inverted microscope to observe their attachment to the media substratum, ensuring their convergence and free of contamination. Every three days culture medium was replaced with a fresh one until cell convergence reached 90 %.

#### 2.3.3 MTT assay

This is a colorimetric assay based on the change of tetrazolium salt (3–(4,5–dimethylthiazol–2–yl)–2,5–diphenyltetrazolium bromide) (MTT) yellow color by NAD(P)H–dependent oxido reductase enzymes in the viable cells to a deep purple color that is insoluble crystalline formazan. According to the MTT kit (Sigma), 20,000 were counted for the cytotoxic test and 5000 cells for the cytostatic. The cells were treated with 100.00 μL of curcumin and curcumin-derived compounds final concentrations under study in each well and incubated for 24 h cytotoxic tests and 72 h for cytostatic tests. After incubation, the media was removed, and all wells were washed with PBS and treated with a 100.00 μL of serum-free RPMI media. The 10.00 μL of MTT solution (0.5 mg/mL) was added to each well, which was incubated for 4 h. Following the removal of the medium and subsequent washing, cells were treated with acidic solution of propanol (100.00 μL of 0.08 N HCl) for 15 min. The absorbance of MTT formazan was determined at 570 nm in a microplate reader (Labtech, UK). Cell viability was determined as the ratio of the treated cells to untreated ones multiplied by 100. Untreated cells in freshly prepared RPMI media were considered as negative control for compounds 1 and 2, while in fresh 1 % DMSO in RPMI media for curcumin.

#### 2.3.4 Statistical analysis

The cytostatic MTT (cell viability data) analyses were performed using GraphPad Prism version 10.2.2 (GraphPad Software, Inc.). For comparisons, “Two-way ANOVA” “Alpha” equal to 0.05 followed by Tukey's multiple comparisons test was applied to determine the statistical significance of the obtained data. Data are expressed as the mean ‡ SD for duplicates. Asterisk denotes significance levels when compared to another group *p < 0.05, **p < 0.01, ***p < 0.001 and ***p < 0.0001 were considered significant while p > 0.05 was considered not significant (ns). The determination of concentration giving maximal 50 % inhibition (IC_50_) by (Interpolated) Restricted cubic spline curve generated by plotting studied concentrations by 32 points were calculated with the X values ranging from 0.001 to 1000 μg/mL using maximum likelihood ratio asymmetrical confidence intervals to standard slope (Hill Slope = « 1.0) was used.

### 2.4 Molecular docking

In this investigation, the protein-ligand blind docking platform CB-Dock [[55], [56], [57], [58]] was employed to assess the interactions between curcumin and the following proteins: MCF-7 breast carcinoma (4MAN) [59] HepG2 liver carcinoma (1HNJ) [60], Colon human cancer (2Y9X), and HeLa cervical carcinoma cell lines (2 × 7F) [61]. CB-Dock is a protein-ligand docking method that automates site identification, adjusts docking box size based on ligands, and uses AutoDock Vina for docking. In general, it improves hit rate and accuracy compared to blind docking. CB-Dock rationalizes the process and enhances accuracy by predicting protein binding sites with CurPocket and ligand binding poses with AutoDock Vina.

## 3 Results and discussion

### 3.1 Design and synthesis of curcumin based azo compounds

**Fig. 2:**
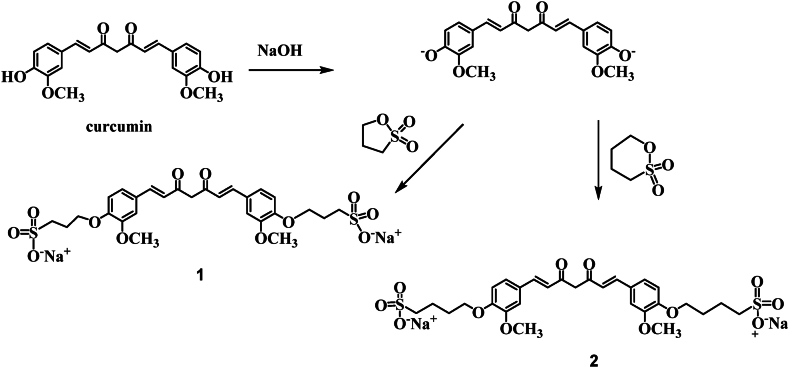
Preparation of curcumin with alkyl sulfonate group.

This study presents a simple procedure for making water-soluble curcumin with alkyl sulfonate moiety. The curcumin sulfonate was prepared by reacting curcumin with 1,3-propane sultone and 1,4-butane sultone as shown in Fig. 2. Curcumin was first converted to alkali curcumin with alkoxide functionality by reacting it with NaOH powder. The produced alkoxide made a nucleophilic attack on sultone, which undergo ring opening to produce the target product in a good yield.

### 3.2 Anticancer activity

Curcumin derivatives compound 1: 3,3'-(((1E,3Z,6E)-3-hydroxy-5-oxohepta-1,3,6-triene-1,7-diyl)bis(6-hydroxy-5-methoxy-3,1phenylene)) bis (propane-1-sulfonic acid), compound 2: 4,4'-(((1E,3Z,6E)-3-hydroxy-5-oxohepta-1,3,6-triene-1,7-diyl)bis(6-hydroxy-5-methoxy-3,1-phenylene))bis(butane-1-sulfonic acid, and curcumin were evaluated for in vitro activity against five selected human cancer cells breast carcinoma (MCF-7), liver carcinoma (HepG2), skin melanoma (B16–F10), Colon human cancer (MDST8) and HeLa cervical carcinoma. Cell viability % after 24 h (cytotoxic) and 72 h (cytostatic) were measured in this study. MTT assay showed variable results related to cancer cell line type, compound type and used concentration in comparison to curcumin and the untreated cells as negative control.

**Fig. 3:**
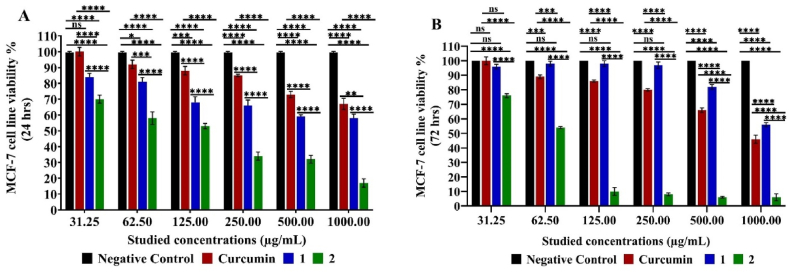
MTT Assay of MCF-7 cell line viability % Cytotoxic (24 h) (**A**) and cytostatic (72 h) (**B**) under the effect of different studied concentrations of compounds 1, 2 and curcumin. Mean values and standard deviation obtained from an average of 20,000 cells for the cytotoxic test (24 h) and 5000 cells for the cytostatic test (72 h) per experiment, total of two experiments for each substance. “Ordinary Two-way ANOVA” “Alpha” equal to 0.05 followed by Tukey's multiple comparisons test to determine the statistical significance of the obtained data, and values of ns *p >0.05;* *p < 0.05, **p < 0.01, ***p < 0.001 and ****p < 0.0001.

Examined compounds 1 and 2 showed varying cytotoxic and cytostatic effects on MCF-7 cell line at different concentrations (31.25–1000.00 μg/mL). They showed cytotoxic effect rather than a cytostatic one recording cell viability % after 24 h lower than after 72 h (84.00 ± 2.26–59.00 ± 1.13 % and 96.00 ± 1.56–82.00 ± 0.56 %, respectively) under compound 1 effect at all examined concentrations except for at 1000.00 μg/mL since cell viability after 24 h (58.00 ± 2.55 %) more than after 72 h (56.00 ± 1.41 %). While, the opposite was recorded under the compound 2 effect being only cytotoxic at 31.25 μg/mL (70.00 ± 2.55 % after 24 h < 76.00±1.41 % after 72 h, respectively) and cytostatic at 62.50–1000.00 μg/mL concentration having 54.00 ± 0.71–6.00 ± 2.40 % after 72 h < 58.00±3.96–17.00 ± 2.69 % after 24 h, respectively, similar to curcumin effect (89.00 ± 1.27–46.00 ± 2.83 % after 72 h < 92.00±2.69–67.00 ± 3.54 % after 24 h, respectively) and at 31.25 μg/mL showed no difference in cell viability after 24 and 72 h. All obtained data were significant (*p < 0.05-****p < 0.0001) in respect to the negative control except for compound 1 cytostatic effect at 31.25–250.00 μg/mL as well as curcumin cytotoxic and cytostatic effect at 31.25 μg/mL were not significant (p > 0.05). Both compounds 1 and 2 showed significant stronger cytotoxic and cytostatic effect than curcumin (**p < 0.01-****p < 0.0001). Despite the higher cytotoxic effect of compound 2 than compound 1, it is associated with significant more cytostatic effect (****p < 0.0001) against MCF-7 cell line at all examined concentrations (Fig. 3).

**Fig. 4:**
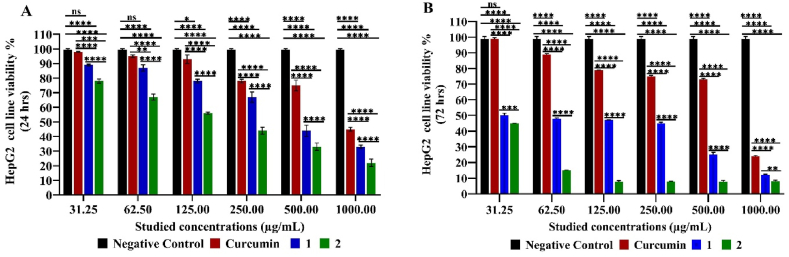
MTT Assay of HepG2 Cell Line Viability % Cytotoxic (24 h) (**A**) and cytostatic (72 h) (**B**) effect under different studied concentrations of compounds 1, 2, and curcumin. Mean values and standard deviation obtained from an average of 20,000 cells for the cytotoxic test (24 h) and 5000 cells for the cytostatic test (72 h) per experiment, total of two experiments for each substance. “Ordinary Two-way ANOVA” “Alpha” equal to 0.05 followed by Tukey's multiple comparisons test to determine the statistical significance of the obtained data, and values of ns *p >0.05;* *p < 0.05, **p < 0.01, ***p < 0.001 and ****p < 0.0001.

While, the effect of compounds 1 and 2 on HepG2 cell line was cytostatic rather than cytotoxic as reduced cell viability % after 72 h (50.00 ± 1.41–12.00 ± 0.42 and 45.00 ± 0.14–8.00 ± 0.71, respectively) more than after 24 h (89.00 ± 0.57–33.00 ± 1.13 and 78.00 ± 1.41–22.00 ± 2.55, respectively) at studied concentration in dose dependent manner, the higher the concentration, the lower cell viability % significantly in comparison to the negative control (****p < 0.0001). As well as achieved by the pure curcumin of being cytostatic (99.00 ± 0.71–24.00 ± 0.42) rather than cytotoxic (98.00 ± 0.28–45.00 ± 1.27) significantly in comparison to the negative control (****p < 0.0001) in concentration dependent manner except at 31.25 μg/mL was not significant (*p >0.05*). Moreover, the cytotoxicity of compounds 1 and 2 was higher than that for curcumin on HepG2 cells (****p < 0.0001). Yet the cytostatic effect of compounds 1 and 2 are far more than curcumin recording cell viability % reduction down to 12.00 ± 0.42, 8.00 ± 0.71 and 24.00 ± 0.42, respectively, out of which compound 2 is the strongest anti-proliferative agent on that cell line as recorded the lowest cell viability (8.00 ± 0.71 at 1000.00 μg/mL) showing significant variations among them (**p < 0.01-****p < 0.0001) (Fig. 4).

**Fig. 5:**
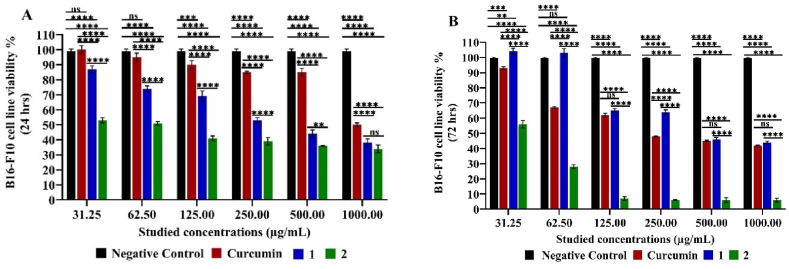
MTT assay of B16–F10 cell cine viability % Cytotoxic (24 h) (**A**) and cytostatic (72 h) (**B**) effect under different studied concentrations of compounds 1, 2 and curcumin. Mean values and standard deviation obtained from an average of 20,000 cells for the cytotoxic test (24 h) and 5000 cells for the cytostatic test (72 h) per experiment, total of two experiments for each substance. “Ordinary Two-way ANOVA” “Alpha” equal to 0.05 followed by Tukey's multiple comparisons test to determine the statistical significance of the obtained data, and values of ns *p >0.05;* *p < 0.05, **p < 0.01, ***p < 0.001 and ****p < 0.0001.

Furthermore, B16–F10 cell line viability was reduced to 38.00 ± 2.55 % and 44.00 ± 0.85 % after 24 and 72 h, respectively at 1000.00 μg/mL in dose depended manner under the effect of compound 1 in a cytotoxic manner significantly in comparison to the negative control (**p < 0.01or ****p < 0.0001) except at 62.50 μg/mL was not significant after 72 h (*p >0.05*). But it was reduced to 6.00 ± 1.27 % and 42.00 ± 0.28 % in a cytostatic manner by compound 2 and curcumin, respectively, at concentrations under study significantly (****p < 0.0001) compared to the untreated B16–F10 cells. However, despite the cytotoxic effect of only compound, both compounds 1 and 2 have more cytotoxicity effect on B16–F 10 cell line than curcumin. Moreover, the highest cytostatic effect against this cell line is by compound 2 (6.00 ± 1.27 %) in comparison to curcumin (42.00 ± 0.28 %) as well as compound 1 (44.00 ± 0.85 %) (****p < 0.0001) (Fig. 5).

**Fig. 6:**
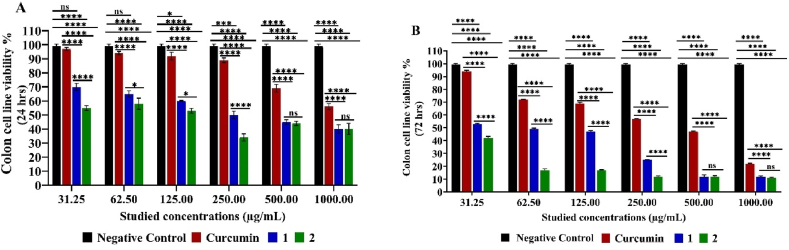
MTT Assay of colon Cell Line Viability % Cytotoxic (24 h) (**A**) and cytostatic (72 h) (**B**) effect under different studied concentrations of compounds 1, 2 and curcumin. Mean values and standard deviation obtained from an average of 20,000 cells for the cytotoxic test (24 h) and 5000 cells for the cytostatic test (72 h) per experiment, total of two experiments for each substance. “Ordinary Two-way ANOVA” “Alpha” equal to 0.05 followed by Tukey's multiple comparisons test to determine the statistical significance of the obtained data, and values of ns *p >0.05;* *p < 0.05, **p < 0.01, ***p < 0.001 and ****p < 0.0001.

The cytotoxic and cytostatic effect assay of compounds 1 and 2 on colon carcinoma cell line revealed a pronounced cytostatic effect (53.00 ± 0.42–12.00 ± 0.57 and 42.00 ± 1.27–11.00 ± 0.42 % cell viability, respectively) with significant performance in comparison to negative control (****p < 0.0001) rather than cytotoxic one (70.00 ± 2.55–40.00 ± 2.97 % cell viability) under the examined concentrations in comparison to curcumin significant (****p < 0.0001) cytostatic one (94.00 ± 0.85–22.00 ± 0.28 cell viability %) in comparison to control. Compound 2 revealed significant better cytostatic effect after 72 h (*p < 0.0001*) than compound 1 except at relatively high concentration 500.00–1000.00 μg/mL) no significant difference (*p >0.05*) (Fig. 6)

**Fig. 7:**
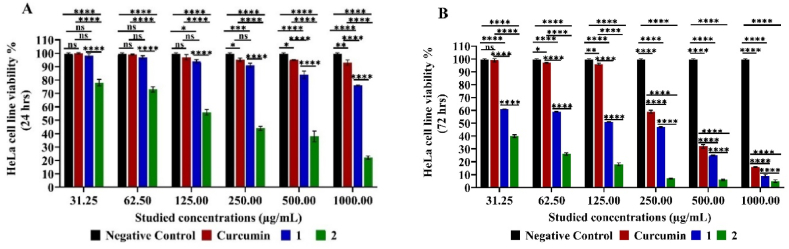
MTT Assay of HeLa Cell Line Viability % Cytotoxic (24 h) (**A**) and cytostatic (72 h) (**B**) effect under different studied concentrations of compounds 1, 2 and curcumin. Mean values and standard deviation obtained from an average of 20,000 cells for the cytotoxic test (24 h) and 5000 cells for the cytostatic test (72 h) per experiment, total of two experiments for each substance. “Ordinary Two-way ANOVA” “Alpha” equal to 0.05 followed by Tukey's multiple comparisons test to determine the statistical significance of the obtained data, and values of ns *p >0.05;* *p < 0.05, **p < 0.01, ***p < 0.001 and ****p < 0.0001.

Similar cytostatic effect was recorded on HeLa cell line as compounds 1 (61.00 ± 0.14–9.00 ± 1.13), compound 2 (78.00 ± 2.40–5.00 ± 0.99) and curcumin (99.00 ± 1.27–16.00 ± 0.28) cell viability % after 72 h relative to the higher cell viability after 24 h (98.00 ± 1.70–76.00 ± 0.42, 78.00 ± 2.40 70–76.00 ± 0.42, respectively) cell viability % in concentration-dependent manner. Obtained data showed higher cell viability. After all, pure curcumin has a lower cytostatic as well as cytotoxic effect than both its derived compounds 1 and 2 on the HeLa cell line significantly (****p<0.0001) except at 31.25–250.00 μg/mL (*p>0.*05). Yet, compound 2 achieved the most pronounced cytostatic effect on that cancer cell line, followed by compound 1 as compared with curcumin (*p < 0.05 or ****p < 0.0001) as shown in Fig. 7.

**Fig. 8:**
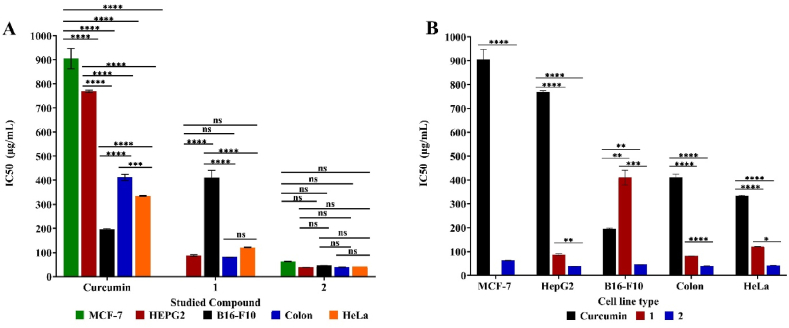
(A and B) IC_50_ values (μg/mL) of the five examined cancer cell lines after 72 h at cell density of 5000 cell/well (cytostatic) using the MTT test under the effect of compounds 1, 2 and curcumin. Mean values and standard deviation obtained from an average of 5000 cells viability % of the cell lines under study per experiment-total of two experiments for each substance. “Ordinary Two-way ANOVA” “Alpha” equal to 0.05 followed by Tukey's multiple comparisons test to determine the statistical significance of the obtained data, and values of ns *p >0.05;* *p < 0.05, **p < 0.01, ***p < 0.001 and ****p < 0.0001.

As tumors grow when cell division (birth) surpasses cell mortality (death), so cryostasis inhibits cell growth and proliferation via the use of cytostatic agents which limit cell growth and division. Therefore, the use of cytostatic drugs in chemotherapy is prevalent, especially for treating cancers. From this point of view, the IC_50_ (Half-maximal inhibitory concentration) of curcumin and the examined derived mainly cytostatic compounds which expected to act as antiproliferative agent rather than direct killing on the different cancer cell lines was calculated to determine their effect under nontoxic cytostatic concentration in μg/mL. Results are summarized in that compound 2 showed the strongest cytostatic effect on MCF-7, HepG2, B16–F10, Colon and HeLa carcinoma cell lines viability % after 72 h with IC50 nontoxic concentrations of 62.46 ± 1.06, 38.41 ± 0.22, 45.62 ± 0.20, 39.86 ± 0.72 and 42.28 ± 0.28 μg/mL, respectively which are lower IC_50_ values than those recorded by the curcumin on the same cancer cell lines under study, respectively (905.06 ± 41.99, 769.26 ± 4.99, 195.28 ± 3.77, 411.89 ± 13.02 and 334.87 ± 1.78 μg/mL) significantly as ****p < 0.0001 for all cancer cell lines under study except for B16–F10 **p < 0.01. Obtained results confirmed the pronounced similar cytostatic effect of compound 1 on all cancer line understudy at almost degree as no significant variation among them despite the different recorded IC50 values (*p >0.05*). Similarly, the derived curcumin compound 1 also has had lower IC50 values 87.89 ± 3.23, 410.50 ± 30.82, 80.99 ± 0.23 and 120.66 ± 1.50 μg/mL than curcumin on HepG2, B16–F10, Colon and HeLa carcinoma cell lines cell lines, respectively, significantly as ****p < 0.0001 for all affected cancer cell lines under study except for B16–F10 **p < 0.01. However, compound 1 did not show significant cytostatic effect on MCF-7, which was after the inability to calculate IC50 value within the studied concentrations range. Moreover, compound 1 showed the higher cytostatic effect on HepG2, Colon and HeLa carcinoma cell lines viability % with no significant variation among them (*p >0.05*), with being B16–F10 the least to be affected in comparison with other affected cell lines (****p < 0.0001). In conclusion, among all the examined compounds, compound 2 showed the strongest cytostatic effect with the lowest IC_50_ concentrations of 38.41 ± 0.22 and 39.86 ± 0.72 μg/mL on both B16–F10 and Colon cancer, respectively. So, the examined cancer lines proliferation was inhibited by the effect of compound 2 and 1 with significant variation among them (*p < 0.05, **p < 0.01, ***p < 0.001 and ****p < 0.0001), except for MCF-7 was not affected by compound 1. So, the recorded cytostatic effect of the investigated compounds on the studied cancer cell lines varied depending on the compound type as well as the cancer cell line type as shown in which is reflected by different IC50 values (Table 1, Fig. 8.).

**Table 1:** IC_50_ values with slandered deviations (μg/mL) of the five examined cancer cell lines after 72 h at cell density of 5000 cell/well (cytostatic) using the MTT test in duplicates under the effect of compounds 1, 2 and curcumin.

Cancer cell Line type	IC50 (μg/mL)			(*p*) 1 vs. Curcumin	(*p*) 2 vs. Curcumin	(p) 1 vs. 2
	Curcumin	1	2			
**MCF-7 breast carcinoma**	905.06 ± 41.99		62.46 ± 1.06		**** (<0.0001)	
**HepG2 liver carcinoma**	769.26 ± 4.99	87.89 ± 3.23	38.41 ± 0.22	**** (<0.0001)	**** (<0.0001)	** (0.0015)
**B16–F10 skin melanoma**	195.28 ± 3.77	410.50 ± 30.82	45.62 ± 0.20	** (0.007)	** (0.007)	*** (0.0006)
**Colon human cancer**	411.89 ± 13.02	80.99 ± 0.23	39.86 ± 0.72	**** (<0.0001)	**** (<0.0001)	**** (<0.0001)
**HeLa cervical carcinoma**	334.87 ± 1.78	120.66 ± 1.50	42.28 ± 0.28	**** (<0.0001)	**** (<0.0001)	* (0.0244)

Mean values and standard deviation obtained from an average of 20,000 and 5000 cells for cytotoxic (24 h) and cytostatic (72 h), respectively, per experimental total of two experiments for each substance. Significance: ns p >0.05; *p < 0.05; **p < 0.01; ***p < 0.001; ****p < 0.0001 (ANOVA, Tukey's post-test).

### 3.3 Molecular docking

Predicting the interactions that take place between proteins and small molecules is an important step in decoding a broad variety of biological processes, as well as an important step in gaining an advantage in drug development [55]. This step plays a significant role in a number of fundamental biological phenomena. Protein–ligand blind docking is an effective method for achieving this goal. This method identifies the areas of a protein responsible for binding and at the same time it predicts the binding positions of a molecule [56]. Recently, there has been an increasing interest in blind docking because enormous protein structures have been determined, opening the doors to the possibility of exploring new target therapies [57]. Due to this, there are now more situations where blind docking is necessary.

In this investigation, the protein-ligand blind docking platform CB-Dock [[57], [58], [59]] was employed to assess the interactions between curcumin and the following proteins: MCF-7 (4MAN) [60] HepG2 (1HNJ) [61], Colon (2Y9X), and HeLa carcinoma cell lines (2 × 7F) [62]. CB-Dock is a protein-ligand docking method that automates site identification, adjusts docking box size based on ligands, and uses Auto Dock Vina for docking. In general, it improves hit rate and accuracy compared to blind docking. CB-Dock rationalizes the process and enhances accuracy by predicting protein binding sites with Cur Pocket and ligand binding poses with Auto Dock Vina.

**Fig. 9:**
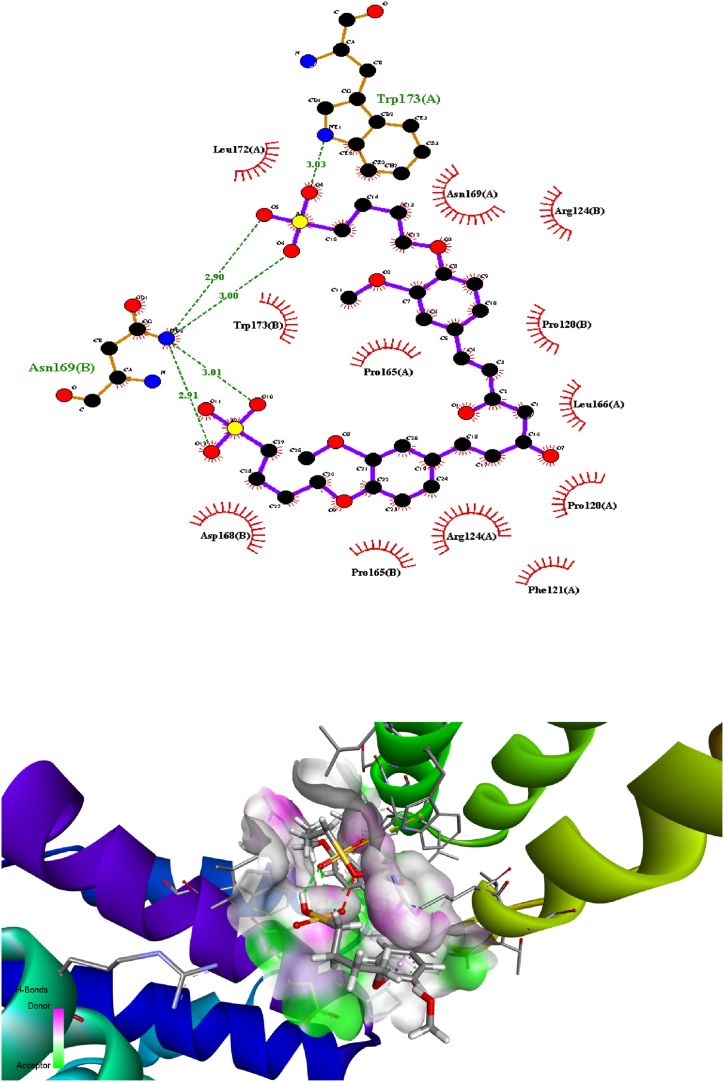

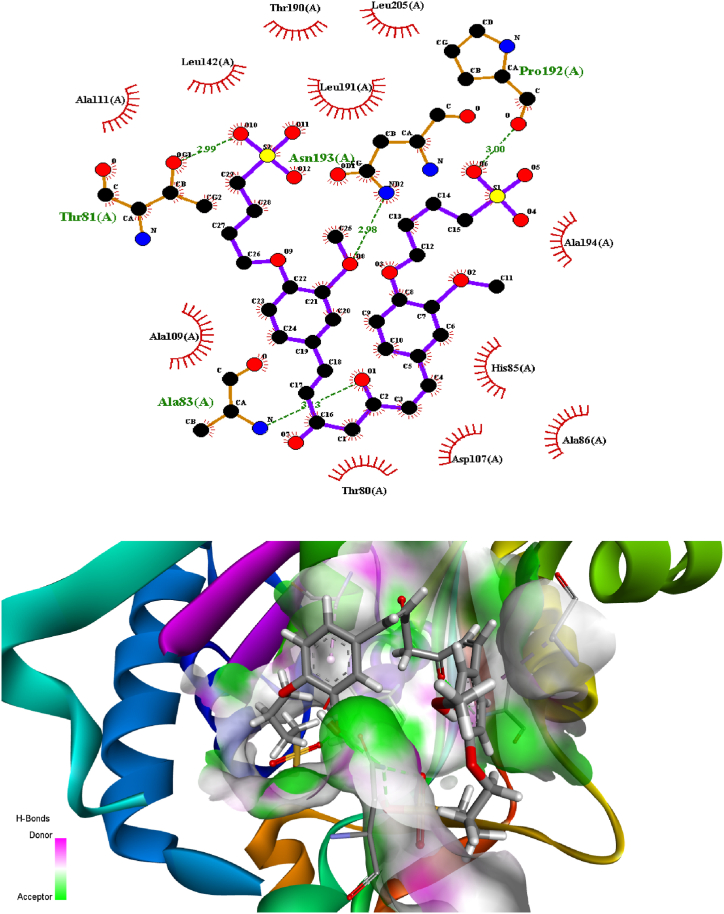

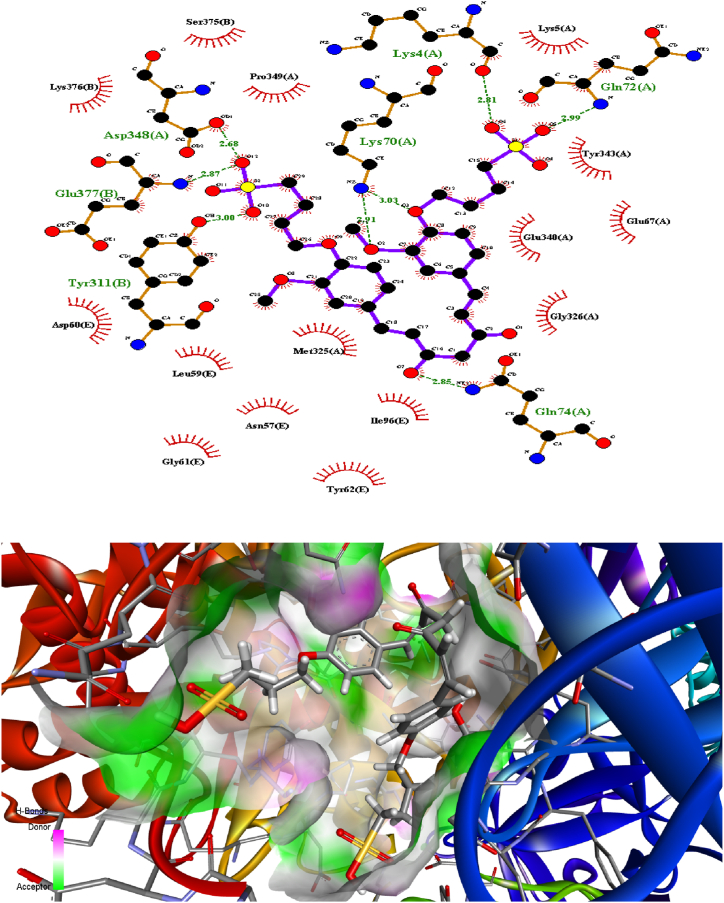

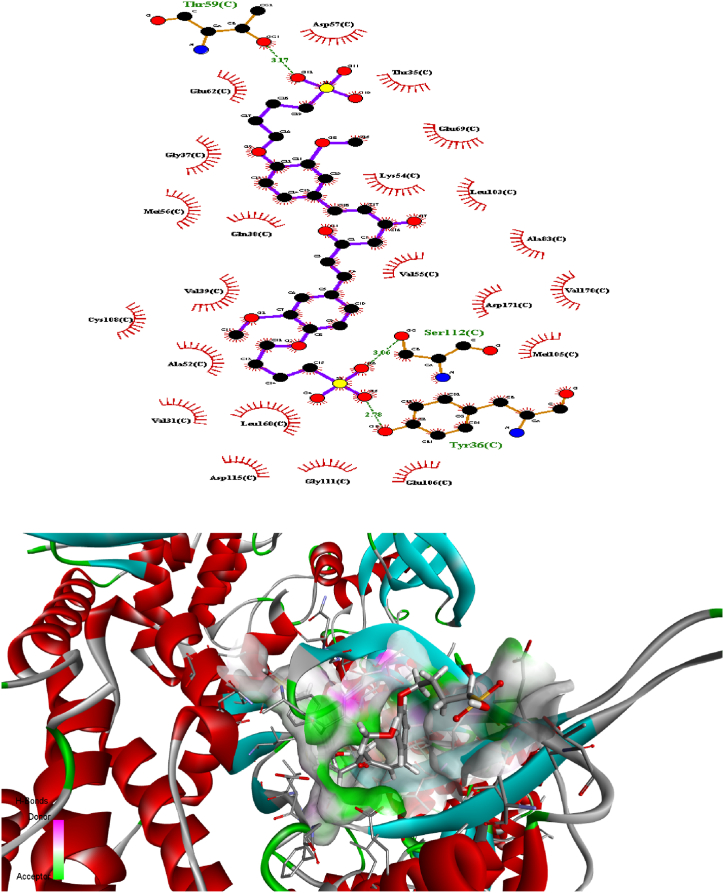
H-bonding docking and the Vina docking score values of **compound 2** with the amino acid side chains of proteins of various cancer cells.

The curcumin molecule is a powerful anticancer candidate drug (as supported by experimental investigation, which exhibited outstanding anticancer activity treatments against many cells), as evidenced by its capacity to form H-bonds during docking with the amino acid side chains and the Vina docking score values as shown in Fig. 9.

## 4 Conclusion

A convenient synthetic method was successfully employed in preparation of a new generation of water-soluble curcumin with alkyl sulfonate moiety. The synthesis was performed in a single step process using available low-cost materials, no special reaction conditions or special equipment were needed. The anticancer activities of the synthesized curcumin alkyl sulfonates were tested against five selected human cancer cells. The cytotoxic and cytostatic anticancer results showed that curcumin alkyl sulfonates have a pronounced cytostatic anticancer effect rather than cytotoxic. The curcumin alkyl sulfonates outperformed curcumin activity against the tested cancer cells and showed to be powerful anticancer candidate drugs as supported by the molecular docking results. The obtained anticancer effect of the curcumin alkyl sulfonate to a certain extent exhibited results that were comparable to those documented in the literature as considered similar cancer types such as skin, liver, colon, and HeLa cell cancer. The results served as the driving force behind the execution of the current study.

## Funding

This research received no funding from any association.

## Additional information

Correspondence and requests for materials should be addressed to Othman Hamed.

## Declarations ethics approval and consent to participate

Not applicable.

## Disclosure statement

The authors declared no potential conflicts of interest concerning the research, authorship, and/or publication of this article.

## Data availability

Not applicable**.** All the relevant data are included in the manuscript. There is no additional data available for this study.

## CRediT authorship contribution statement

**Alaa Janem:** Methodology. **Ghader Omar:** Data curation. **Othman Hamed:** Writing – original draft, Supervision, Conceptualization. **Shehdeh Jodeh:** Writing – review & editing. **Abdalhadi Deghles:** Investigation. **Avni Berisha:** Software. **Waseem Mansour:** Visualization. **Saber Abu Jabal:** Formal analysis. **Oswa Fares:** Data curation. **Ataa Jaser:** Investigation. **Ameed Amireh:** Resources. **Ghaleb Adwan:** Resources.

## Declaration of competing interest

The authors declare that they have no known competing financial interests or personal relationships that could have appeared to influence the work reported in this paper.
