# Supplementary material for: Water soluble curcumin with alkyl sulfonate moiety: Synthesis, and anticancer efficacy
Source: Heliyon. 2024 Jun 28;10(13):e33808. doi: 10.1016/j.heliyon.2024.e33808 (PMC11261864; doi:10.1016/j.heliyon.2024.e33808)
Supplement: Multimedia component 1 [file mmc1.docx]

**WATER SOLUBLE CURCUMIN WITH ALKYLSULFONATE MOIETY: SYNTHESIS, AND ANTICANCER EFFICACY**

Alaa Janem^1^, Ghader Omar^2*^, Othman Hamed^1*^, Shehdeh Jodeh^1*^, Abdalhadi Deghles^3^, Avni Berisha^4,5^, Waseem Mansour^1^, Saber Abu Jabal^1^, Oswa Fares^1^, Ataa Jaser^1^, Ameed Amireh^1^, Ghaleb Adwan^2^

^1^Chemistry Department, Faculty of Science, An-Najah National University, P.O. Box 7, Nablus Palestine. (O.H.) [ohamed@najah.edu](mailto:ohamed@najah.edu).

^2^Biology Department, Faculty of Sciences An-Najah National University, P.O. Box 7, Nablus Palestine. (G.O.) [ghaderomar@najah.edu](mailto:ghaderomar@najah.edu)

^3^Department of chemistry^,^ Istiqlala University, Jericho, Palestine

^4^ Department of Chemistry, Faculty of Natural and Mathematics Science, University of Prishtina, Prishtina 10000 Kosovo.

^5^Materials Science-Nanochemistry Research Group, Nano Alb-Unit of Albanian Nanoscience and Nanotechnology, Tirana 1000 Albania.


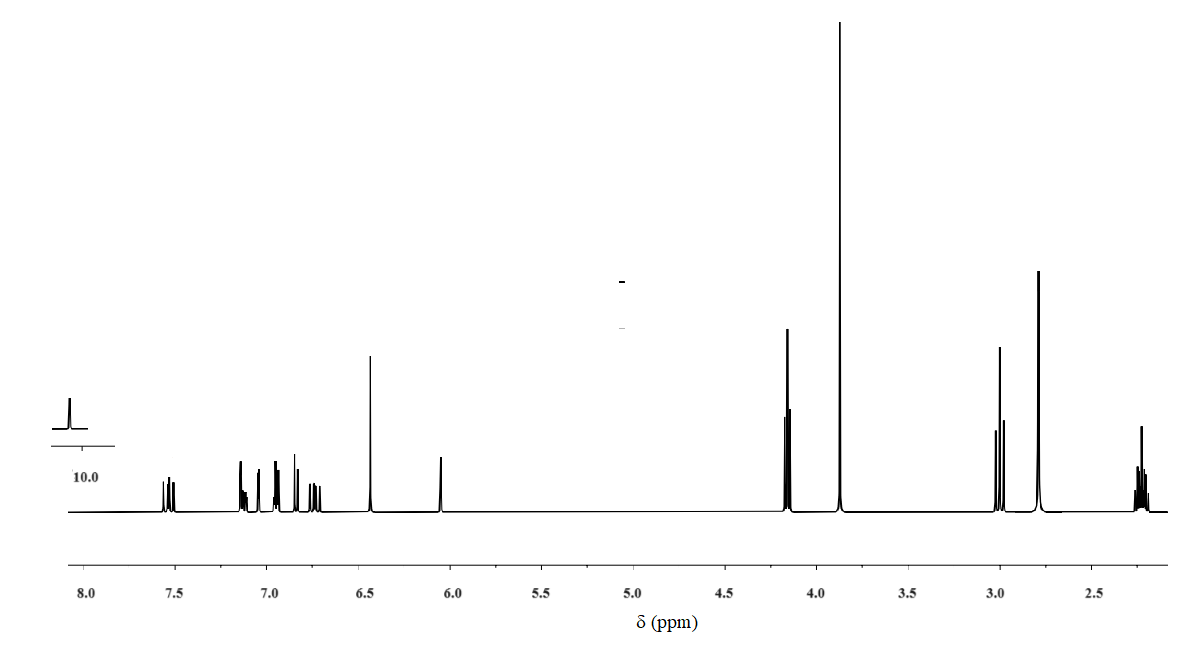

Supplementary Figure 1. ^1^H NMR of 3,3'-(((1E,3Z,6E)-3-hydroxy-5-oxohepta-1,3,6-triene-1,7-diyl)bis(6-hydroxy-5-methoxy-3,1phenylene)) bis (propane-1-sulfonic acid) **(1)**

**
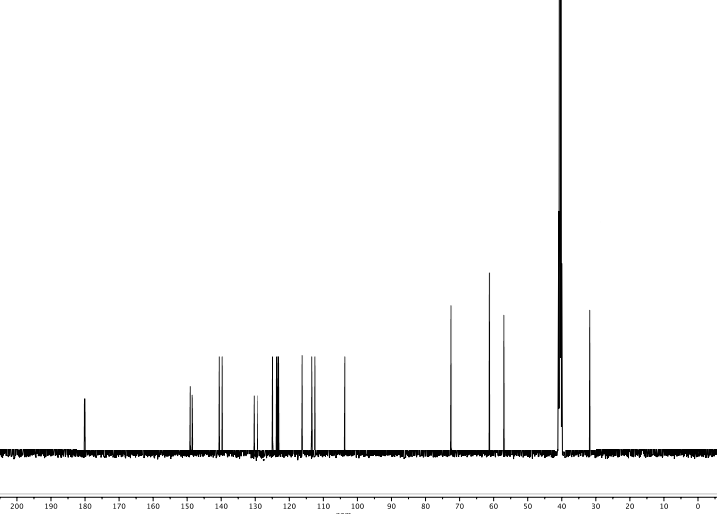
**

**Supplementary Figure 2.** C-13 NMR of 3,3'-(((1E,3Z,6E)-3-hydroxy-5-oxohepta-1,3,6-triene-1,7-diyl)bis(6-hydroxy-5-methoxy-3,1phenylene)) bis (propane-1-sulfonic acid) **(1)**


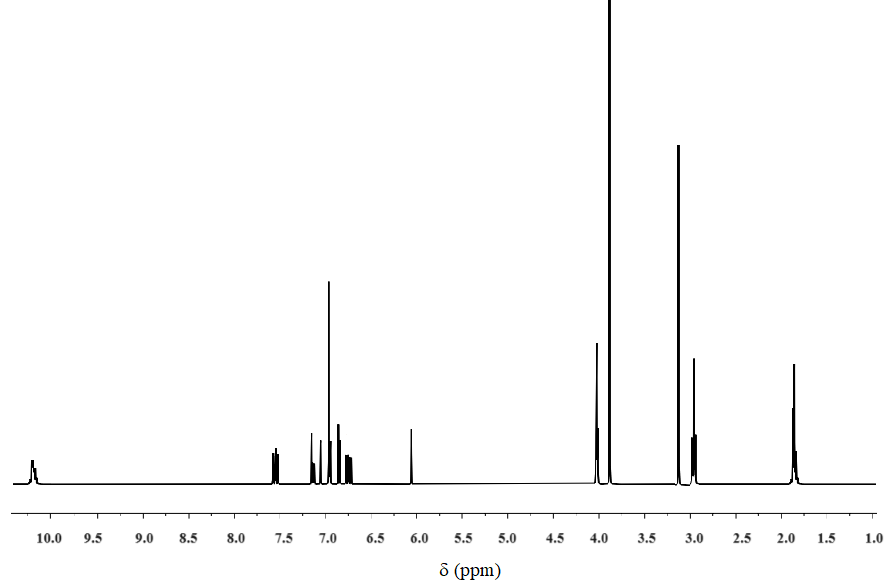

Supplementary Figure 3. ^1^H NMR of 4,4'-(((1E,3Z,6E)-3-hydroxy-5-oxohepta-1,3,6-triene-1,7-diyl)bis(6-hydroxy-5-methoxy-3,1-phenylene))bis(butane-1-sulfonic acid **(2)**

**
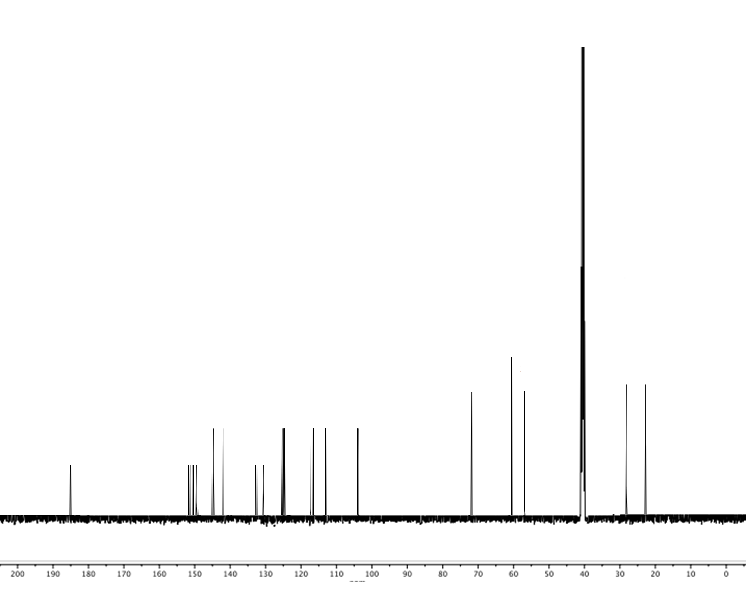
**

Supplementary Figure 4**.** C-13 NMR of 4,4'-(((1E,3Z,6E)-3-hydroxy-5-oxohepta-1,3,6-triene-1,7-diyl)bis(6-hydroxy-5-methoxy-3,1-phenylene))bis(butane-1-sulfonic acid **(2)**

Supplementary Table I. MTT assay of MCF-7 cell line viability % cytotoxic (24 h) and cytostatic (72 h) under the effect of different studied concentrations of compounds; 1: 3,3'-(((1E,3Z,6E)-3-hydroxy-5-oxohepta-1,3,6-triene-1,7-diyl)bis(6-hydroxy-5-methoxy-3,1-phenylene))bis(propane-1-sulfonic acid), 2: 4,4'-(((1E,3Z,6E)-3-hydroxy-5-oxohepta-1,3,6-triene-1,7-diyl)bis(6-hydroxy-5-methoxy-3,1-phenylene))bis(butane-1-sulfonic acid) and curcumin in comparison to each other and to negative control.

| **Studied concentrations (µg/mL)** | **Cytotoxic (24 h) Cytostatic (72 h)** | **Curcumin** | | **1** | | | **2** | | | **(*p*)**  **1**  **vs.**  **2** |
| --- | --- | --- | --- | --- | --- | --- | --- | --- | --- | --- |
|  |  | **Cell viability %** | **(*p*)**  **vs. Negative Control** | **Cell viability %** | **(*p*)**  **1 vs. Negative Control** | **(*p*)**  **1 vs.  Curcumin** | **Cell viability %** | **(*p*)**  **2 vs. Negative Control** | **(*p*)**  **2 vs.  Curcumin** |  |
| **31.25** | **24 h** | 100.00 ± 2.69 | p > 0.05 | 84.00 ± 2.26 | p < 0.0001 | p < 0.0001 | 70.00 ± 2.55 | p < 0.0001 | p < 0.0001 | p < 0.0001 |
|  | **72 h** | 100.00 ± 2.69 | p > 0.05 | 96.00 ± 1.56 | p > 0.05 | p > 0.05 | 76.00 ± 1.41 | p < 0.0001 | p < 0.0001 | p < 0.0001 |
| **62.50** | **24 h** | 92.00 ± 2.69 | p < 0.05 | 81.00 ± 2.69 | p < 0.0001 | p < 0.001 | 58.00 ± 3.96 | p < 0.0001 | p < 0.0001 | p < 0.0001 |
|  | **72 h** | 89.00 ± 1.27 | p < 0.001 | 98.00 ± 1.70 | p > 0.05 | p < 0.001 | 54.00 ± 0.71 | p < 0.0001 | p < 0.0001 | p < 0.0001 |
| **125.00** | **24 h** | 88.00 ± 2.69 | p < 0.001 | 68.00 ± 3.54 | p < 0.0001 | p < 0.0001 | 53.00 ± 1.70 | p < 0.0001 | p < 0.0001 | p < 0.0001 |
|  | **72 h** | 86.00 ± 0.71 | p < 0.0001 | 98.00 ± 1.98 | p > 0.05 | p < 0.0001 | 10.00 ± 2.69 | p < 0.0001 | p < 0.0001 | p < 0.0001 |
| **250.00** | **24 h** | 85.00 ± 0.71 | p < 0.0001 | 66.00 ± 3.54 | p < 0.0001 | p < 0.0001 | 34.00 ± 2.55 | p < 0.0001 | p < 0.0001 | p < 0.0001 |
|  | **72 h** | 80.00 ± 0.85 | p < 0.0001 | 97.00 ± 2.26 | p > 0.05 | p < 0.0001 | 8.00 ± 0.99 | p < 0.0001 | p < 0.0001 | p < 0.0001 |
| **500.00** | **24 h** | 73.00 ± 1.98 | p < 0.0001 | 59.00 ± 1.13 | p < 0.0001 | p < 0.0001 | 32.00 ± 2.55 | p < 0.0001 | p < 0.0001 | p < 0.0001 |
|  | **72 h** | 66.00 ± 1.56 | p < 0.0001 | 82.00 ± 1.56 | p < 0.0001 | p < 0.0001 | 6.00 ± 0.57 | p < 0.0001 | p < 0.0001 | p < 0.0001 |
| **1000.00** | **24 h** | 67.00 ± 3.54 | p < 0.0001 | 58.00 ± 2.55 | p < 0.0001 | p < 0.01 | 17.00 ± 2.69 | p < 0.0001 | p < 0.0001 | p < 0.0001 |
|  | **72 h** | 46.00 ± 2.83 | p < 0.0001 | 56.00 ± 1.41 | p < 0.0001 | p < 0.001 | 6.00 ± 2.40 | p < 0.0001 | p < 0.0001 | p < 0.0001 |

Mean values and standard deviation obtained from an average of 20,000 cells for the cytotoxic test (24 h) and 5,000 cells for the cytostatic test (72 h) per experiment, of two experiments for each compound. "Ordinary Two-way ANOVA" "Alpha" = 0.05 followed by Tukey's multiple comparisons test to determine the statistical significance of the obtained data

Supplementary Table II. MTT Assay of HepG2 cell line viability % cytotoxic (24 h) and cytostatic (72 h) under the effect of different studied concentrations of compounds; 1: 3,3'-(((1E,3Z,6E)-3-hydroxy-5-oxohepta-1,3,6-triene-1,7-diyl)bis(6-hydroxy-5-methoxy-3,1-phenylene))bis(propane-1-sulfonic acid), 2: 4,4'-(((1E,3Z,6E)-3-hydroxy-5-oxohepta-1,3,6-triene-1,7-diyl)bis(6-hydroxy-5-methoxy-3,1-phenylene))bis(butane-1-sulfonic acid) and curcumin in comparison to each other and to negative control.

| **Studied concentrations (µg/mL)** | **Cytotoxic (24 h) Cytostatic (72 h)** | **Curcumin** | | **1** | | | **2** | | | **(*p*)**  **1**  **vs.**  **2** |
| --- | --- | --- | --- | --- | --- | --- | --- | --- | --- | --- |
|  |  | **Cell viability %** | **(*p*)**  **vs. Negative Control** | **Cell viability %** | **(*p*)**  **1 vs. Negative Control** | **(*p*)**  **1 vs.  Curcumin** | **Cell viability %** | **(*p*)**  **2 vs. Negative Control** | **(*p*)**  **2 vs.  Curcumin** |  |
| **31.25** | **24 h** | 98.00 ± 0.28 | p > 0.05 | 89.00 ± 0.57 | p < 0.0001 | p < 0.001 | 78.00 ± 1.41 | p < 0.0001 | p < 0.0001 | p < 0.0001 |
|  | **72 h** | 99.00 ± 0.71 | p > 0.05 | 50.00 ± 1.41 | p < 0.0001 | p < 0.0001 | 45.00 ± 0.14 | p < 0.0001 | p < 0.0001 | p < 0.001 |
| **62.50** | **24 h** | 95.00 ± 0.85 | p > 0.05 | 87.00 ± 2.26 | p < 0.0001 | p < 0.01 | 67.00 ± 2.12 | p < 0.0001 | p < 0.0001 | p < 0.0001 |
|  | **72 h** | 89.00 ± 0.57 | p < 0.0001 | 48.00 ± 0.42 | p < 0.0001 | p < 0.0001 | 15.00 ± 0.28 | p < 0.0001 | p < 0.0001 | p < 0.0001 |
| **125.00** | **24 h** | 93.00 ± 2.83 | p < 0.05 | 78.00 ± 1.27 | p < 0.0001 | p < 0.0001 | 56.00 ± 0.71 | p < 0.0001 | p < 0.0001 | p < 0.0001 |
|  | **72 h** | 79.00 ± 0.14 | p < 0.0001 | 47.00 ± 0.14 | p < 0.0001 | p < 0.0001 | 7.70 ± 0.71 | p < 0.0001 | p < 0.0001 | p < 0.0001 |
| **250.00** | **24 h** | 78.00 ± 1.27 | p < 0.0001 | 67.00 ± 3.54 | p < 0.0001 | p < 0.0001 | 44.00 ± 2.40 | p < 0.0001 | p < 0.0001 | p < 0.0001 |
|  | **72 h** | 75.00 ± 0.57 | p < 0.0001 | 45.00 ± 0.71 | p < 0.0001 | p < 0.0001 | 7.80 ± 0.28 | p < 0.0001 | p < 0.0001 | p < 0.0001 |
| **500.00** | **24 h** | 75.00 ± 3.68 | p < 0.0001 | 44.00 ± 3.82 | p < 0.0001 | p < 0.0001 | 33.00 ± 2.55 | p < 0.0001 | p < 0.0001 | p < 0.0001 |
|  | **72 h** | 73.00 ± 0.71 | p < 0.0001 | 25.00 ± 1.56 | p < 0.0001 | p < 0.0001 | 7.60 ± 0.99 | p < 0.0001 | p < 0.0001 | p < 0.0001 |
| **1000.00** | **24 h** | 45.00 ± 1.27 | p < 0.0001 | 33.00 ± 1.13 | p < 0.0001 | p < 0.0001 | 22.00 ± 2.55 | p < 0.0001 | p < 0.0001 | p < 0.0001 |
|  | **72 h** | 24.00 ± 0.42 | p < 0.0001 | 12.00 ± 0.42 | p < 0.0001 | p < 0.0001 | 8.00 ± 0.71 | p < 0.0001 | p < 0.0001 | p < 0.01 |

Mean values and standard deviation obtained from an average of 20,000 cells for the cytotoxic test (24 h) and 5,000 cells for the cytostatic test (72 h) per experiment, of two experiments for each compound. "Ordinary Two-way ANOVA" "Alpha" = 0.05 followed by Tukey's multiple comparisons test to determine the statistical significance of the obtained data

Supplementary Table III. MTT assay of B16-F10 cell line viability % cytotoxic (24 h) and cytostatic (72 h) under the effect of different studied concentrations of compounds; 1: 3,3'-(((1E,3Z,6E)-3-hydroxy-5-oxohepta-1,3,6-triene-1,7-diyl)bis(6-hydroxy-5-methoxy-3,1-phenylene))bis(propane-1-sulfonic acid), 2: 4,4'-(((1E,3Z,6E)-3-hydroxy-5-oxohepta-1,3,6-triene-1,7-diyl)bis(6-hydroxy-5-methoxy-3,1-phenylene))bis(butane-1-sulfonic acid) and curcumin in comparison to each other and to negative control.

| **Studied concentrations (µg/mL)** | **Cytotoxic (24 h) Cytostatic (72 h)** | **Curcumin** | | **1** | | | **2** | | | **(*p*)**  **1**  **vs.**  **2** |
| --- | --- | --- | --- | --- | --- | --- | --- | --- | --- | --- |
|  |  | **Cell viability %** | **(*p*)**  **vs. Negative Control** | **Cell viability %** | **(*p*)**  **1 vs. Negative Control** | **(*p*)**  **1 vs.  Curcumin** | **Cell viability %** | **(*p*)**  **2 vs. Negative Control** | **(*p*)**  **2 vs.  Curcumin** |  |
| **31.25** | **24 h** | 100.00 ± 2.69 | p > 0.05 | 87.00 ± 2.26 | p < 0.0001 | p < 0.0001 | 53.00 ± 1.70 | p < 0.0001 | p < 0.0001 | p < 0.0001 |
|  | **72 h** | 93.00 ± 0.85 | p < 0.001 | 104.00 ± 2.12 | p < 0.01 | p < 0.0001 | 56.00 ± 2.40 | p < 0.0001 | p < 0.0001 | p < 0.0001 |
| **62.50** | **24 h** | 95.00 ± 2.69 | p > 0.05 | 74.00 ± 1.98 | p < 0.0001 | p < 0.0001 | 51.00 ± 1.13 | p < 0.0001 | p < 0.0001 | p < 0.0001 |
|  | **72 h** | 67.00 ± 0.57 | p < 0.0001 | 103.00 ± 2.97 | p > 0.05 | p < 0.0001 | 28.00 ± 1.27 | p < 0.0001 | p < 0.0001 | p < 0.0001 |
| **125.00** | **24 h** | 90.00 ± 2.69 | p < 0.001 | 69.00 ± 3.54 | p < 0.0001 | p < 0.0001 | 41.00 ± 1.56 | p < 0.0001 | p < 0.0001 | p < 0.0001 |
|  | **72 h** | 62.00 ± 1.27 | p < 0.0001 | 65.00 ± 1.41 | p < 0.0001 | p > 0.05 | 7.00 ± 1.27 | p < 0.0001 | p < 0.0001 | p < 0.0001 |
| **250.00** | **24 h** | 85.00 ± 0.71 | p < 0.0001 | 53.00 ± 1.70 | p < 0.0001 | p < 0.0001 | 39.00 ± 2.55 | p < 0.0001 | p < 0.0001 | p < 0.0001 |
|  | **72 h** | 48.00 ± 0.28 | p < 0.0001 | 64.00 ± 1.56 | p < 0.0001 | p < 0.0001 | 6.00 ± 0.28 | p < 0.0001 | p < 0.0001 | p < 0.0001 |
| **500.00** | **24 h** | 85.00 ± 2.55 | p < 0.0001 | 44.00 ± 2.55 | p < 0.0001 | p < 0.0001 | 36.00 ± 0.28 | p < 0.0001 | p < 0.0001 | p < 0.01 |
|  | **72 h** | 45.00 ± 0.42 | p < 0.0001 | 46.00 ± 1.27 | p < 0.0001 | p > 0.05 | 6.00 ± 1.56 | p < 0.0001 | p < 0.0001 | p < 0.0001 |
| **1000.00** | **24 h** | 50.00 ± 1.27 | p < 0.0001 | 38.00 ± 2.55 | p < 0.0001 | p < 0.0001 | 34.00 ± 2.55 | p < 0.0001 | p < 0.0001 | p > 0.05 |
|  | **72 h** | 42.00 ± 0.28 | p < 0.0001 | 44.00 ± 0.85 | p < 0.0001 | p > 0.05 | 6.00 ± 1.27 | p < 0.0001 | p < 0.0001 | p < 0.0001 |

Mean values and standard deviation obtained from an average of 20,000 cells for the cytotoxic test c(24 h) and 5,000 cells for the cytostatic test (72 h) per experiment, total of two experiments for each substance. "Ordinary Two-way ANOVA" "Alpha" equal to 0.05 followed by Tukey's multiple comparisons test to determine the statistical significance of the obtained data

Supplementary Table IV. MTT Assay of colon cell line viability % cytotoxic (24 h) and cytostatic (72 h) under the effect of different studied concentrations of compounds; 1: 3,3'-(((1E,3Z,6E)-3-hydroxy-5-oxohepta-1,3,6-triene-1,7-diyl)bis(6-hydroxy-5-methoxy-3,1-phenylene))bis(propane-1-sulfonic acid), 2: 4,4'-(((1E,3Z,6E)-3-hydroxy-5-oxohepta-1,3,6-triene-1,7-diyl)bis(6-hydroxy-5-methoxy-3,1-phenylene))bis(butane-1-sulfonic acid) and curcumin in comparison to each other and to negative control.

| **Studied concentrations (µg/mL)** | **Cytotoxic (24 h) Cytostatic (72 h)** | **Curcumin** | | **1** | | | **2** | | | **(*p*)**  **1**  **vs.**  **2** |
| --- | --- | --- | --- | --- | --- | --- | --- | --- | --- | --- |
|  |  | **Cell viability %** | **(*p*)**  **vs. Negative Control** | **Cell viability %** | **(*p*)**  **1 vs. Negative Control** | **(*p*)**  **1 vs.  Curcumin** | **Cell viability %** | **(*p*)**  **2 vs. Negative Control** | **(*p*)**  **2 vs.  Curcumin** |  |
| **31.25** | **24 h** | 97.00 ± 1.13 | p > 0.05 | 70.00 ± 2.55 | p < 0.0001 | p < 0.0001 | 55.00 ± 1.70 | p < 0.0001 | p < 0.0001 | p < 0.0001 |
|  | **72 h** | 94.00 ± 0.85 | p < 0.0001 | 53.00 ± 0.42 | p < 0.0001 | p < 0.0001 | 42.00 ± 1.27 | p < 0.0001 | p < 0.0001 | p < 0.0001 |
| **62.50** | **24 h** | 94.00 ± 1.27 | p > 0.05 | 65.00 ± 2.12 | p < 0.0001 | p < 0.0001 | 58.00 ± 3.96 | p < 0.0001 | p < 0.0001 | p < 0.05 |
|  | **72 h** | 72.00 ± 0.14 | p < 0.0001 | 49.00 ± 0.71 | p < 0.0001 | p < 0.0001 | 17.00 ± 0.99 | p < 0.0001 | p < 0.0001 | p < 0.0001 |
| **125.00** | **24 h** | 92.00 ± 2.69 | p < 0.05 | 60.00 ± 0.28 | p < 0.0001 | p < 0.0001 | 53.00 ± 1.70 | p < 0.0001 | p < 0.0001 | p < 0.05 |
|  | **72 h** | 69.00 ± 0.85 | p < 0.0001 | 47.00 ± 0.85 | p < 0.0001 | p < 0.0001 | 17.00 ± 0.28 | p < 0.0001 | p < 0.0001 | p < 0.0001 |
| **250.00** | **24 h** | 89.00 ± 1.70 | p < 0.001 | 50.00 ± 2.69 | p < 0.0001 | p < 0.0001 | 34.00 ± 2.55 | p < 0.0001 | p < 0.0001 | p < 0.0001 |
|  | **72 h** | 57.00 ± 0.14 | p < 0.0001 | 25.00 ± 0.14 | p < 0.0001 | p < 0.0001 | 12.00 ± 0.57 | p < 0.0001 | p < 0.0001 | p < 0.0001 |
| **500.00** | **24 h** | 69.00 ± 2.69 | p < 0.0001 | 45.00 ± 1.56 | p < 0.0001 | p < 0.0001 | 44.00 ± 1.56 | p < 0.0001 | p < 0.0001 | p > 0.05 |
|  | **72 h** | 47.00 ± 0.42 | p < 0.0001 | 12.00 ± 1.27 | p < 0.0001 | p < 0.0001 | 12.00 ± 0.71 | p < 0.0001 | p < 0.0001 | p > 0.05 |
| **1000.00** | **24 h** | 56.00 ± 2.12 | p < 0.0001 | 40.00 ± 2.97 | p < 0.0001 | p < 0.0001 | 40.00 ± 3.96 | p < 0.0001 | p < 0.0001 | p > 0.05 |
|  | **72 h** | 22.00 ± 0.28 | p < 0.0001 | 12.00 ± 0.57 | p < 0.0001 | p < 0.0001 | 11.00 ± 0.42 | p < 0.0001 | p < 0.0001 | p > 0.05 |

Mean values and standard deviation obtained from an average of 20,000 cells for the cytotoxic test c(24 h) and 5,000 cells for the cytostatic test (72 h) per experiment, total of two experiments for each substance. "Ordinary Two-way ANOVA" "Alpha" equal to 0.05 followed by Tukey's multiple comparisons test to determine the statistical significance of the obtained data

Supplementary Table V. HeLa Cell line viability % cytotoxic (24 h) and cytostatic (72 h) under the effect of different studied concentrations of compounds; 1: 3,3'-(((1E,3Z,6E)-3-hydroxy-5-oxohepta-1,3,6-triene-1,7-diyl)bis(6-hydroxy-5-methoxy-3,1-phenylene))bis(propane-1-sulfonic acid), 2: 4,4'-(((1E,3Z,6E)-3-hydroxy-5-oxohepta-1,3,6-triene-1,7-diyl)bis(6-hydroxy-5-methoxy-3,1-phenylene))bis(butane-1-sulfonic acid) and curcumin in comparison to each other and to negative control.

| **Studied concentrations (µg/mL)** | **Cytotoxic (24 h) Cytostatic (72 h)** | **Curcumin** | | **1** | | | **2** | | | **(*p*)**  **1**  **vs.**  **2** |
| --- | --- | --- | --- | --- | --- | --- | --- | --- | --- | --- |
|  |  | **Cell viability %** | **(*p*)**  **vs. Negative Control** | **Cell viability %** | **(*p*)**  **1 vs. Negative Control** | **(*p*)**  **1 vs.  Curcumin** | **Cell viability %** | **(*p*)**  **2 vs. Negative Control** | **(*p*)**  **2 vs.  Curcumin** |  |
| **31.25** | **24 h** | 100.00 ± 0.42 | p > 0.05 | 98.00 ± 1.70 | p > 0.05 | p > 0.05 | 78.00 ± 2.40 | p < 0.0001 | p < 0.0001 | p < 0.0001 |
|  | **72 h** | 99.00 ± 1.27 | p > 0.05 | 61.00 ± 0.14 | p < 0.0001 | p < 0.0001 | 40.00 ± 1.13 | p < 0.0001 | p < 0.0001 | p < 0.0001 |
| **62.50** | **24 h** | 99.00 ± 0.42 | p > 0.05 | 97.00 ± 1.13 | p > 0.05 | p > 0.05 | 73.00 ± 1.98 | p < 0.0001 | p < 0.0001 | p < 0.0001 |
|  | **72 h** | 97.00 ± 0.42 | p < 0.05 | 59.00 ± 0.28 | p < 0.0001 | p < 0.0001 | 26.00 ± 0.99 | p < 0.0001 | p < 0.0001 | p < 0.0001 |
| **125.00** | **24 h** | 97.00 ± 1.98 | p > 0.05 | 94.00 ± 1.27 | p < 0.05 | p > 0.05 | 56.00 ± 2.12 | p < 0.0001 | p < 0.0001 | p < 0.0001 |
|  | **72 h** | 96.00 ± 0.71 | p < 0.01 | 51.00 ± 0.14 | p < 0.0001 | p < 0.0001 | 18.00 ± 1.13 | p < 0.0001 | p < 0.0001 | p < 0.0001 |
| **250.00** | **24 h** | 95.00 ± 1.27 | p < 0.05 | 91.00 ± 1.56 | p < 0.001 | p > 0.05 | 44.00 ± 1.56 | p < 0.0001 | p < 0.0001 | p < 0.0001 |
|  | **72 h** | 59.00 ± 1.13 | p < 0.0001 | 47.00 ± 0.42 | p < 0.0001 | p < 0.0001 | 7.00 ± 0.42 | p < 0.0001 | p < 0.0001 | p < 0.0001 |
| **500.00** | **24 h** | 95.00 ± 0.28 | p < 0.05 | 84.00 ± 2.69 | p < 0.0001 | p < 0.0001 | 38.00 ± 3.96 | p < 0.0001 | p < 0.0001 | p < 0.0001 |
|  | **72 h** | 32.00 ± 1.41 | p < 0.0001 | 25.00 ± 0.42 | p < 0.0001 | p < 0.0001 | 6.00 ± 0.57 | p < 0.0001 | p < 0.0001 | p < 0.0001 |
| **1000.00** | **24 h** | 93.00 ± 1.98 | p < 0.01 | 76.00 ± 0.42 | p < 0.0001 | p < 0.0001 | 22.00 ± 1.27 | p < 0.0001 | p < 0.0001 | p < 0.0001 |
|  | **72 h** | 16.00 ± 0.28 | p < 0.0001 | 9.00 ± 1.13 | p < 0.0001 | p < 0.0001 | 5.00 ± 0.99 | p < 0.0001 | p < 0.0001 | p < 0.001 |

Mean values and standard deviation obtained from an average of 20,000 cells for the cytotoxic test c(24 h) and 5,000 cells for the cytostatic test (72 h) per experiment, total of two experiments for each substance. "Ordinary Two-way ANOVA" "Alpha" equal to 0.05 followed by Tukey's multiple comparisons test to determine the statistical significance of the obtained data
